# The hereditary angioedema burden of illness study in Europe (HAE-BOIS-Europe): background and methodology

**DOI:** 10.1186/1471-5945-12-4

**Published:** 2012-04-26

**Authors:** Anette Bygum, Emel Aygören-Pürsün, Teresa Caballero, Kathleen Beusterien, Shadi Gholizadeh, Patience Musingarimi, Suzanne Wait, Henrik Boysen

**Affiliations:** 1 HAE Centre Denmark, Department of Dermatology and Allergy Centre, Odense University Hospital, 5000 Odense C, Denmark; 2 Department of Pediatrics, Pediatric Hematology, Oncology, Hemostaseology and Cardiology, University Hospital, Johann Wolfgang Goethe University, Theodor-Stern-Kai 7, 60596 Frankfurt, Germany; 3 Allergy Department, Hospital La Paz Health Research Center (IdiPaz), Biomedical Research Network on Rare Diseases U754 (CIBERER), University Hospital La Paz, Paseo de la Castellana 261, 28046 Madrid, Spain; 4 Oxford Outcomes Inc, 7315 Wisconsin Ave. Ste 250W, Bethesda, MD 20814 USA; 5 ViroPharma, Chatsworth House, 29 Broadway, Maidenhead, SL6 1LY, UK; 6 SHW Health Ltd, 40 Lena Gardens, London, W6 7PZ, UK; 7 HAEi–International Patient Organization for C1 Inhibitor Deficiencies, Lindeparken 33, DK-6230, Roedekro, Denmark

## Abstract

**Background:**

Hereditary angioedema (HAE) is a rare but serious disease marked by swelling attacks in the extremities, face, trunk, airway, or abdominal areas that can be spontaneous or the result of trauma and other triggers. It can be life-threatening due to the risk of asphyxiation. While there have been major advancements in our understanding of the immunogenetics of HAE, there are significant gaps in the literature regarding understanding of the humanistic and economic impact of the disease, particularly in Europe. The purpose of the HAE Burden of Illness Study-Europe (HAE-BOIS-Europe), the development and methodology of which is described here, is to better understand the management and impact of HAE from the patient perspective in Europe.

**Methods/Design:**

This is a cross-sectional study in which retrospective data were also collected being conducted in Denmark, Germany and Spain. The study is open to patients ages 12 and older with a diagnosis of HAE-I or HAE-II. Data collection includes: (i) a survey on individuals’ health care resource use, direct and indirect medical costs, impact on work and school, treatment satisfaction, and emotional functioning (via the Hospital Anxiety and Depression Scale); and (ii) one-on-one interviews to collect detailed descriptive data and patient testimonials on the impact of HAE on patients’ health-related quality of life.

**Discussion:**

The present manuscript describes the development and plans for implementing a multi-country European study with the aim of characterizing the humanistic and economic burden of HAE from the patient perspective. This study will help raise awareness of HAE as a rare but debilitating condition with wide-ranging impacts.

## Background

**Figure 1:**
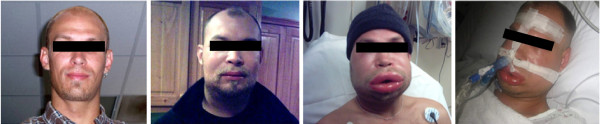
HAE patient experiencing HAE attacks.

Hereditary angioedema (HAE) is a rare but serious autosomal dominant disorder marked by swelling attacks in the extremities, face, trunk, airways, or abdominal areas that can be spontaneous or the result of trauma [1] (Figure 1). Attacks can be serious; the risk of dying from airway obstruction, if left untreated, has been estimated at 30 % [2,3]. Attack frequency is described in the literature as ranging from rarely to once every 3 days [4]. Untreated, patients on average experience attacks every one to two weeks [5].

The prevalence of HAE is estimated to be approximately 1 in every 50,000 persons, with no marked differences among ethnic groups [6,7]. The rarity of the disease, together with the fact that 6.1–13.7 % of patients may be asymptomatic or have delayed symptom onset [7-9], means that, while HAE symptoms often begin in early childhood and persist throughout patients’ lives, awareness of the condition is extremely low, and diagnosis is frequently delayed. As observed in a Danish study, the mean delay to diagnosis can be as great as 16 years [7]. There is therefore an urgent need to raise awareness of the disease and its appropriate diagnosis and treatment among clinicians and families who have a hereditary predisposition to the condition.

Improvements in our understanding of the disease process have led to the recent availability of an increased range of treatment options for HAE. Disease management takes the form of treatment of acute attacks, short-term prophylactic treatment for the prevention of attacks (for example, before a surgical procedure), and long-term prophylactic treatment (prevention) to minimize the frequency and severity of ongoing attacks [5,10,11]. Treatments currently approved in Europe for acute attacks include plasma derived (pd) C1-inhibitor [human] concentrate (Berinert, Cetor), pd nano-filtered C1-inhibitor [human] (Cinryze), recombinant C1-inhibitor (Ruconest/Rhucin), and use of a bradykinin B2 receptor antagonist (Firazyr). Long-term prophylaxis (prevention) options have traditionally involved androgens (such as danazol or stanozolol), although their approval status for HAE varies among European countries. Cinryze is the only agent to have received European approval for both the treatment and pre-procedure prevention of angioedema attacks in adults and adolescents with HAE, as well as for the routine prevention of angioedema attacks in adults and adolescents.

The mechanisms underlying what prompts HAE attacks to start and resolve are relatively unknown. Moreover, the severity of symptoms is highly variable both from one patient to another and within a given patient [5]. The uncertainty surrounding the onset of an attack can cause great anxiety for patients given that the swelling—especially when affecting the airways—can be fatal. HAE may, therefore, have a substantial emotional impact on the patient as well as on his or her family. In a cross-sectional survey using the Hamilton Depression Inventory – Short Form questionnaire (HDI-SF), 42.5 % of HAE patients had scores indicative of depressive symptomatology [12]. Such findings suggest the importance of considering potential mental health impacts like stress, in addition to traditional treatment, in the clinical management of these patients in order to reduce the burden of HAE on daily life.

Some studies, including both interventional and case studies, have explored the health-related quality of life (HRQoL) impacts of HAE. Interventional studies have used general health status measures like the SF-36 and SF-12, and the Dermatology Life Quality Index (DLQI), which focuses on dermatological quality of life issues, and have found them to be associated with improvements in several quality of life areas, including both physical and psychological parameters [13-16]. However, authors noted limitations of the DLQI, as it was originally created for use in patients with chronic dermatological diseases with exacerbations such as psoriasis and refers to symptoms that may not be relevant for HAE. Also, it does not take acute attacks into consideration and therefore does not allow to measure patients’ HRQoL both during an attack and in between attacks.

The SF-36 and SF-12 and general HRQoL measures are useful for making comparisons across disease areas, but they may fail to capture specific HRQoL manifestations of HAE. As Lumry and colleagues (2010) showed, HAE was associated with detriments in HRQoL across physical and mental health domains and in each subscale of both the SF-12 and HDI-SF [12]. However, without in-depth elaboration from patients, the interpretation of these impacts, particularly those that are primarily physical, is limited. Second, while depression was assessed by way of the HDI-SF, anxiety, arguably an important emotional marker for any chronic condition marked by sudden attacks, was not captured in this measure. Prior and colleagues are developing an international multi-language HAE-specific HRQoL measure for adults, the IHAE-QoL [17,18]. The pilot study has been completed in 12 countries (Argentina, Austria, Brazil, Canada, Denmark, France, Germany, Hungary, Israel, Poland, Romania, and Spain) and the psychometric study is being performed in order to validate this instrument (T Caballero, personal communication). This disease-specific questionnaire is expected to give more detailed and relevant data on HRQoL in HAE patients in the years ahead. However, this measure is, as of the publishing of the present article, still unavailable.

**Figure 2:**
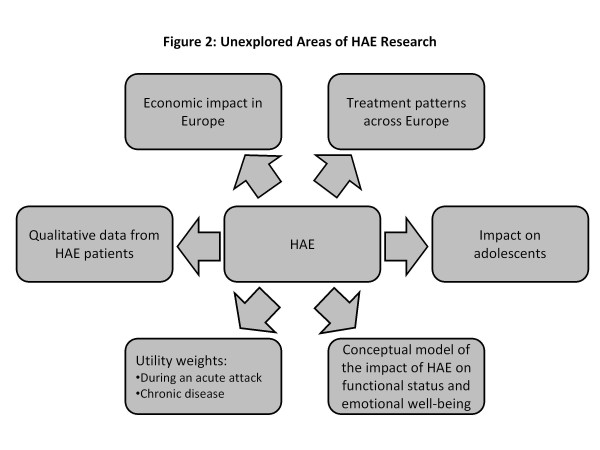
Gaps in the literature with regard to the humanistic and economic burden of HAE.

While there have been advances in our understanding of the burden of illness in HAE, significant gaps in our knowledge remain, particularly with regards to the humanistic and economic burden of the disease (Figure 2). There is a dearth of data on: the economic impact of HAE; comparisons of HAE treatment patterns, patient characteristics, and outcomes in different countries; the impact of HAE on adolescents; and qualitative research, including interviews and/or focus group studies with HAE patients. In addition, utility weights (preference values) have not been published for HAE. Such weights may be used to quality-adjust life expectancy for use in economic evaluations of medical interventions. Finally, no-one has yet developed a conceptual model visually depicting the relationships between HAE disease symptoms and more distal impacts, such as impact on career advancement and educational attainment.

There is also a critical need for better epidemiological data on HAE generally. The data from this study will help further inform such data. In addition, the findings likely can be compared with those from other studies, including HAE registries, such as the Sweha-Reg [19], the European Register of HAE [20], and observational studies such as the HAE nationwide survey conducted in Denmark [7].

Given the aforementioned gaps in our knowledge of HAE, the objective of the HAE Burden of Illness Study-Europe (HAE-BOIS-Europe) is to characterize the humanistic and economic burden of HAE from the patient perspective. This large-scale, scientifically robust, multi-country European study evaluates the real-world experience of HAE patients with respect to resource utilization and HRQoL burden of HAE in Germany, Denmark, and Spain. It collects qualitative data in addition to quantitative data to support a conceptual model of the patient-perceived impacts of HAE on HRQoL, showing the quality of life and economic burden of HAE both in relation to acute attacks as well as the long-term experience of HAE. The present paper describes the development and plans for implementation of this multi-country European study, the initial findings of which will be publicly available in 2012.

## Methods

**Figure 3:**
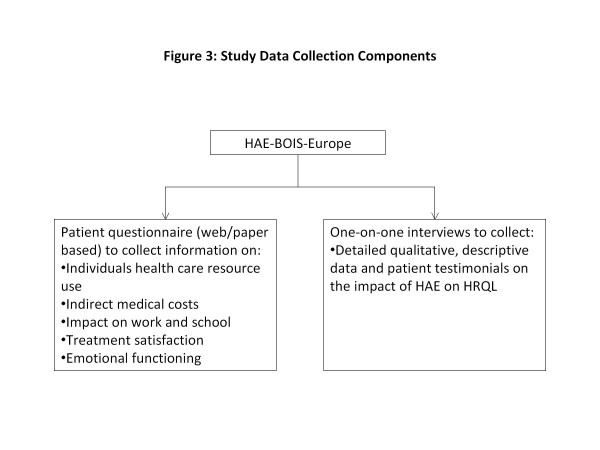
Study data collection components.

The HAE-BOIS-Europe is a cross-sectional study in which retrospective data were also collected conducted in Denmark, Germany and Spain, with participating patients being recruited from HAE centres of excellence, as well as HAE patient associations in each country. The two data collection components include a web- or paper-based questionnaire and one-on-one telephone interviews, designed to collect both quantitative and qualitative data from patients as illustrated in Figure 3. To be eligible for inclusion in the study, patients must have a diagnosis of HAE-I or HAE-II (with C-1 inhibitor deficiency), be aged 12 years or older, have had at least one attack in the past six months, and demonstrate adequate fluency in the language in which they are taking the survey/interview. Patients with HAE-III or acquired angioedema are not eligible for inclusion into the study.

### Ethics review and patient confidentiality

Prior to study recruitment, the protocol, including the questionnaire and interview guide, were translated into the native language of each target country and submitted to the ethics review board of each Centre as per the particular requirements of each Centre. The elements of country-specific regulations pertaining to consent procedures, disclosure of potential risks and benefits and subject confidentiality have been strictly observed. All study subjects are required to read and endorse a consent form (for participants aged 12–17, parents endorse the consent form and adolescents then endorse an assent form before data collection).

For the individual telephone interviews, the study coordinator screens patients, and those eligible and interested are scheduled for an anonymous telephone interview to be conducted by an interviewer trained in qualitative research by the research company Oxford Outcomes. All interviewers are fluent in the native language of the country in which the interview is taking place.

### Recruitment

The principal investigators at the HAE Centres of Excellence in Frankfurt, Germany (Klinikum der Johann Wolfgang Goethe-Universität Frankfurt), Odense, Denmark (Odense Universitetshospital), and Madrid, Spain (Hospital Universitario La Paz) have contacted potentially eligible patients via mail, email or phone and/or advertised the study at the respective sites. In addition, the HAE patient associations in Denmark, Germany, and Spain have advertised the study on their website and/or have contacted members in their database. Contact has been made via phone, mail, or email with individuals who have noted that they would like to be contacted for HAE-related studies. The recruitment process has been designed to minimize selection bias. Specifically, in Denmark, almost all HAE patients have been invited; in Germany and Spain, all potentially eligible patients in the patient organization database, and a random sample of patients from the HAE Centers of Excellence, were invited.

Consistent with other burden of illness studies of rare conditions in Europe [21,22], the target sample size is 150. This sample size is sufficient to identify key impacts of HAE. The plan is to include equal numbers (n = 50) from each country, ensuring that each country-specific sample was generalizable through random sampling. For the telephone interviews, the target sample size is 30 patients with HAE (10 per country). Previous qualitative research has found that after twelve interviews, between 88 and 92 % of themes can be identified [23].

**Figure 4:**
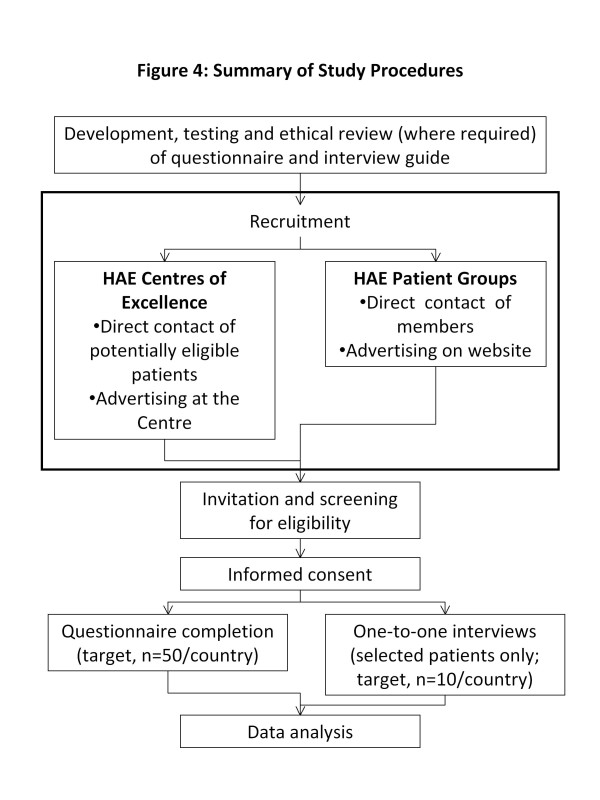
Summary of study procedures.

The invitation letter includes a username and password used to access the survey; this allows individuals to return to the survey and complete it in multiple sittings. Patients who access the web link are screened online in the initial questions, and are led automatically to the consent forms and web survey. Patients without internet access call the study coordinator for a telephone screening; if the patient is eligible and interested, the study coordinator obtains the contact information to send the paper based consent form and survey. A summary of the study procedures is illustrated in Figure 4.

### Instruments

**Figure 5:**
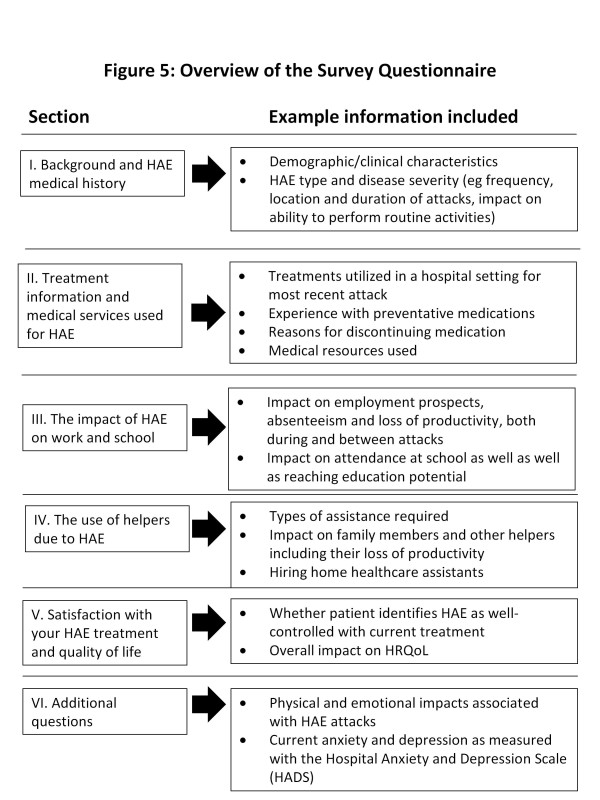
Overview of study questionnaire.

The questionnaire is comprised of six sections intended to assess healthcare resource use and indirect costs. Examples of the data collected in each section are illustrated in Figure 5. The last section includes assessment of current anxiety and depression using the Hospital Anxiety and Depression Scale (HADS), a validated 14 item scale to assess anxiety and depression [24]. The HADS was developed specifically to identify the likelihood of ‘caseness (or confirmed diagnostic condition)’ with respect to anxiety disorders and depression among patients in non-psychiatric settings [24].

The one-on-one interviews are conducted using a semi-structured interview guide. The questions are largely open-ended, allowing the patient to discuss all the various ways that HAE impacts his/her life, such as what is it like to live with HAE, how HAE affects day-to-day activities, and impact of HAE on school and work advancement. Participants are also asked socio-demographic questions during the telephone interviews. Selected questions are the following:

· What is it like to live with HAE?

· Since being diagnosed with HAE, has your life changed? In what ways?

· What aspect of HAE is the most burdensome?

· When was your last HAE attack?

Can you describe what this was like?

Could you tell that you were about to have an attack?

How much did your attack affect your activities? In what way?

· What does it feel like between attacks?

· How does having HAE affect your daily life?

Does it affect your social life?

Does it affect your relationships?

Does it affect your ability to work or attend school?

Does this differ based on location of the attack?

· Are there any symptoms of HAE other than the attacks that impact your life in any way?

The surveys were pilot tested in Denmark, Spain, and Germany with nine participants who met the eligibility criteria and provided feedback on the survey. The pilot testers provided valuable feedback such as clarifying wording in certain questions, but no major changes to the surveys were required or proposed. In general, patients who took the pilot test found the survey understandable and highly relevant to their disease.

### Analysis

Table 1 summarises the major anticipated analyses from HAE-BOIS-Europe. Descriptive analyses, including means, 95 % confidence intervals, and percentages, will be used to describe the medical resource utilization and work productivity variables in the survey. The Hospital Anxiety and Depression Scale (HADS) will be scored as recommended by the developer [24].

**Table 1:** Summary of the major anticipated analyses of data from HAE-BOIS-Europe

**Data source**	**Analyses**
Survey data	Descriptive analyses, eg. · Treatments used · Medical resource utilization · Impact on work and school productivity · Reliance on volunteer and paid helpers · HADS scores and other HRQoL estimates
	Potential categorizations of HAE severity (frequency, pain, duration, etc)
	Estimated health utility weights for: · An acute HAE attack – based on survey responses mapped to the EQ-5D · Chronic disease – based on HADS scores mapped to the EQ-5D
Interviews	Key concepts important to patients with HAE · Providing context to survey-derived quantitative findings · Providing input into the draft conceptual model of HAE burden of illness

The analyses conducted in the HAE-BOIS-Europe study will yield such benefits as the potential to:

· Gain a comprehensive understanding of the humanistic and the direct and indirect resource impacts of HAE from the European patient perspective

· Provide feedback to patients and their families on the patient community experience with HAE

· Help advocate and raise awareness of HAE among policymakers and the global community

· Help raise awareness of the full impact of HAE among the clinical community and contribute to professional education on HAE

This study also will provide an opportunity to explore different categorizations of HAE severity, for example, using attack frequency, duration of swelling, worst pain associated with attack, etc. and their associations with medical resource utilization and impact on ability to perform daily activities. Such analyses may help elucidate how well selected definitions of disease severity differentiate among functional and resource utilization impacts and may help inform clinical management decisions. Recent international consensus guidelines on HAE management, for example, do not provide detail on assessment of disease severity [10].

In addition, the study may provide data that can be used to estimate health utility (preference) weights for HAE. Health utilities are weights scaled from 0.0 (death) to 1.0 (full health). Such weights are used in cost-effectiveness evaluations to quality-adjust life expectancy, thereby producing quality-adjusted life years (QALYs) for medical interventions. Although the HAE-BOIS-Europe study does not directly collect utility data, we plan to estimate utilities for having an acute HAE attack as well as for chronic disease (in between attacks) using the survey data. Specifically, to estimate utilities for an acute attack, we plan to map response items from the burden of illness survey to corresponding items of the EQ-5D, a widely used utility questionnaire. These burden of illness items inquire about worst pain during the most recent attack, the duration that the attack prevented daily activities, the extent that the attack affected ability to perform regular activities, and anxiety about future attacks. We will map the responses to these items to the respective items within the EQ-5D and arrive at an estimated utility for an acute attack. The EQ-5D is a standardised instrument for use as a measure of health outcome that is applicable to a wide range of health conditions and is recommended by the UK’s National Institute for Health and Clinical Excellence (NICE) [25]. A similar methodology will be used to estimate utilities for chronic HAE (in between attacks).

With respect to the one-on-one interviews, the interview scripts will be evaluated to identify key impacts of HAE and to provide context for enhancing interpretation of the survey findings. In qualitative research, there are no power calculations to determine sample size; rather, interviews should continue until saturation—that is, the point at which no new information is being obtained—is reached. In general, saturation is attained by the 10^th^ or 12^th^ interview. The findings will help substantiate and refine the conceptual model on the burden of illness of HAE.

### Draft conceptual model

**Figure 6:**
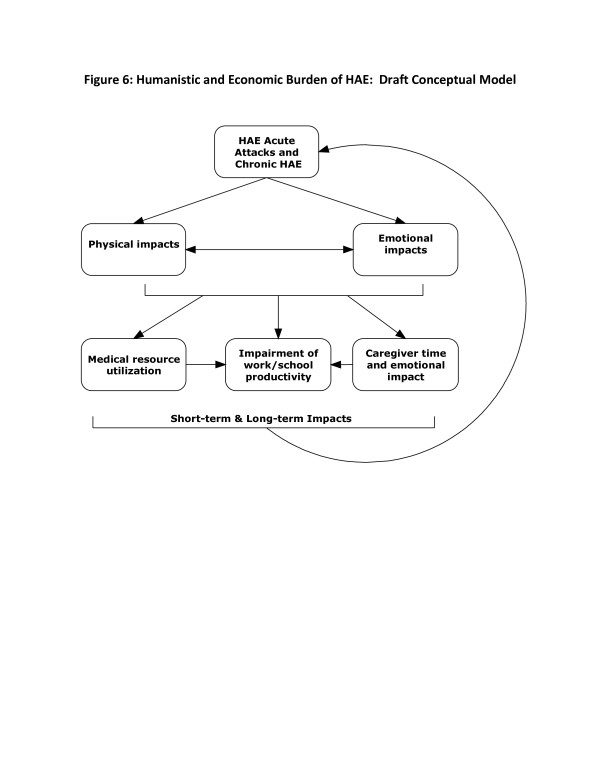
Humanistic and economic burden of HAE: draft conceptual model.

Conceptual models draw on a number of theories and concepts to help understand a particular problem in a specific population. Specifically, a conceptual model diagram shows relationships among a set of concepts such as disease symptoms, treatment, outcomes, and patient perceptions [26,27]. Conceptual models integrate biological and psychological aspects of health outcomes and can help to better understand the patient experience with a disease and specific areas that could benefit from treatment. Figure 6 shows a draft of such a conceptual model for HAE that illustrates both proximal as well as long-term impacts of HAE. This model was developed based on the initial review of the literature; it will be refined based on data from the current study.

## Discussion and conclusion

As with other rare conditions, knowledge of HAE can be limited even among medical professionals. There is therefore a need to collect and understand data describing the clinical, economic, and HRQoL impact of the disorder. While there has been a trend toward trying to better understand the burden of illness in HAE in recent years, a number of key gaps in the literature remain that have prevented a comprehensive understanding of the real-life experience of patients living with HAE and their families. A 2010 study by Wilson and colleagues [28] explored costs associated with HAE in the US and showed a considerable economic burden to patients, payers, and society at large. A study by Lumry *et al.* (2010) discussed humanistic impacts in the same sample but qualitative interviews with patients were not conducted [12]. Thus a study that includes both an economic evaluation and in-depth qualitative research in Europe would be a useful addition to the literature.

Our study will build upon the US study [12,28], however, aspects of it will be more robust. For example, our study will: include adolescents; include patients recruited from patient organizations as well as medical centres; undergo ethics review at clinical sites to ensure data protection and sound methodology; include individual qualitative patient interviews; be conducted in parallel in three countries; and provide data on the humanistic burden in addition to the economic burden of illness. Further, it is hoped that the present study will provide a more comprehensive picture of the overall impact of HAE in that it will evaluate the impact both during acute HAE attacks and in-between attacks. The study will also allow the evaluation of any differences in outcomes for patients who report that their disease is well-controlled as opposed to those who consider their disease as poorly controlled. Ultimately, this study should help to better understand the impact of HAE from the patient perspective and may be very valuable in order to increase awareness of the impact of HAE on individuals and the respective health care systems in which HAE individuals are treated.

In conclusion, the HAE-BOIS Europe study is as the first study of its kind in Europe to obtain a comprehensive picture of HAE from the patient perspective. Its findings should help make HAE more visible in the European and global community, providing evidence for rational stakeholder discussion and informed interventions to help improve the lives of patients living with HAE.

## Competing interests

This research was funded by ViroPharma SPRL.

Anette Bygum, MD has been involved in clinical research or educational events involving CSL Behring, Jerini/Shire, Sobi and ViroPharma.

Emel Aygören-Pürsün, MD has received sponsorship for educational purposes and has provided consultancy services or has participated in clinical trials sponsored by CSL-Behring, Jerini AG/Shire, Sobi and ViroPharma.

Teresa Caballero, MD has received sponsorship for educational purposes, has been paid for providing consultancy services, or has taken part in clinical trials sponsored by Jerini AG/Shire, CSL-Behring, Pharming NV, and ViroPharma.

Kathleen Beusterien and Shadi Gholizadeh work for Oxford Outcomes Inc., an ICON Company, which consults for ViroPharma.

Patience Musingarimi receives consulting fees from ViroPharma.

Suzanne Wait PhD receives consulting fees from ViroPharma.

Henrik Boysen is the Executive Director of HAEi - International Patient Organization for C1 Inhibitor Deficiencies, which receives funding from most pharmaceutical companies, including ViroPharma, the sponsor of this study.

## Authors’ contributions

All authors contributed to the study design. KB and SG drafted the manuscript, and all authors provided substantive input to the manuscript. All authors read and approved the final manuscript.

## Authors’ information

AB: Associate professor, HAE Centre Denmark, Department of Dermatology and Allergy Centre, Odense University Hospital, 5000 Odense C, Denmark

EAP: Specialist for Internal Medicine and Haemostaseology, Department of Pediatrics, University Hospital Frankfurt, Germany; Member of the World Allergy Association (WAO) - HAE International Alliance Steering Committee

TC: Allergy specialist; HAE expert; Consultant at Allergy Department from University Hospital La Paz, Madrid, Spain; Member of SEAIC, EAAACI, AAAAI; Coordinator of GEAB/SGAB (Spanish Group for the study of Angioedema induced by Bradykinin) within SEAIC (Spanish Society of Allergy and Clinical Immunology), researcher of the Biomedical Research Network on Rare Diseases U754 (CIBERER).

KB and SG: Consultants working at Oxford Outcomes, a subsidiary of ICON plc.

PM: Patience Musingarimi is a healthcare consultant currently working for ViroPharma.

SW: Suzanne Wait PhD is a health policy consultant and Director of SHW Health Ltd. She is also Senior Research Fellow at University College London.

HB: Henrik Boysen is the Executive Director of HAEi - International Patient Organization for C1 Inhibitor Deficiencies and the President of the Danish HAE patient organization. He was diagnosed with HAE (type I) at the age of 17 years.

## Pre-publication history

The pre-publication history for this paper can be accessed here:

http://www.biomedcentral.com/1471-5945/12/4/prepub
